# Subretinal echinococcosis: a case report

**DOI:** 10.1186/s12886-017-0581-5

**Published:** 2017-10-05

**Authors:** Chunying Guo, Ruilin Zhu, Jianxing Qiu, Lina Zhu, Liu Yang

**Affiliations:** 1 Department of Ophthalmology, Peking University First Hospital, Key Laboratory of Vision Loss and Restoration, Ministry of Education, No. 8 Xi Shi Ku Street, Xicheng District, Beijing, 100034 China; 2 Department of Radiology, Peking University First Hospital, Beijing, 100034 China

**Keywords:** Echinococcosis, Parasitic disease, Panuveitis, Vitrectomy

## Abstract

**Background:**

Echinococcosis is a dangerous zoonotic parasitic disease. Ocular echinococcosis is very rare, especially the hydatid cysts in subretinal space. We present a case of subretinal echinococcosis and management.

**Case presentation:**

A 37-year-old man with subretinal echinococcosis who developed panuveitis and visual impairment. The patient lives on agriculture and animal husbandry, which made him susceptible to parasitic infection. He had severe panuveitis and blurred vision on arrival at hospital. According to his ocular examination and systemic review, the subretinal echinococcosis diagnosis was made. The patient received pars plana lensectomy and pars plana vitrectomy. The lesion underneath his retina was removed, and histopathology examination confirmed the subretinal echinococcosis diagnosis.

**Conclusions:**

Echinococcosis is a dangerous zoonotic parasitic disease in pastoral areas. Ocular echinococcosis is usually secondary to systemic infection. Although the incidence is rare, the disease could lead to destructive visual function impairment.

## Background

Echinococcosis, also called hydatid disease or hydatidosis, is considered as one of the most dangerous zoonotic parasitic disease worldwide, mainly distributing in Mediterranean regions, Russia, central Asia, China, Australia, South America, and north and east Africa [1, 2]. The liver and the lungs are the common affected organs. Ocular echinococcosis is very rare, especially the hydatid cysts in subretinal space. Here we presented a case of a 37-year-old man with subretinal echinococcosis and treated successfully by operation.

## Case presentation

A 37-year-old man presented with blurring of vision and blocking of visual field in his left eye, associated with eye redness and pain for 40 days. The patient was a Tibetan who lived on agriculture and animal husbandry. There was a history of contact with cattle and dogs. Systemic history was unremarkable.

**Fig. 1:**
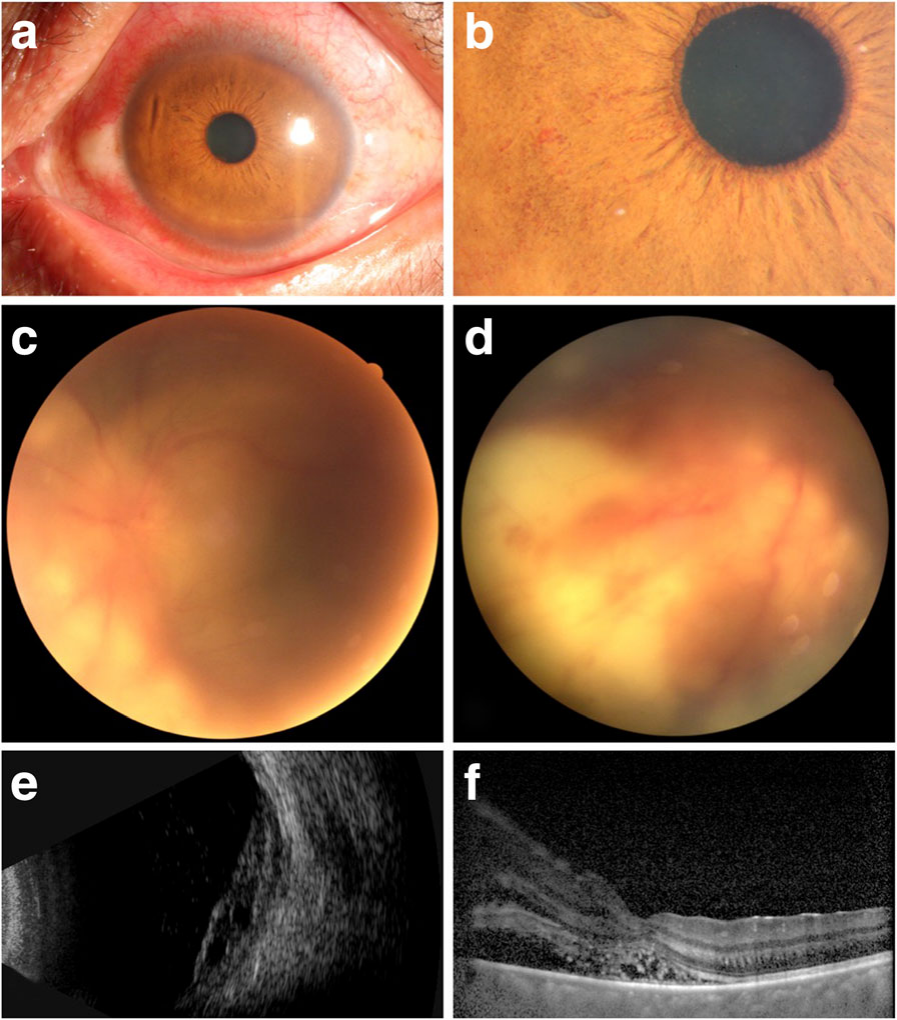
Anterior segment photography of the patient showed the mixed congestion of the eye (**a**), and iris neovascularization near the pupillary margin (**b**). Fundus photography showed the nasal retinal detachment and whitish mass lesions underneath the retina (**c**, **d**). Ultrasonography revealed retinal detachment and subretinal cystic lesions (**e**). SD-OCT showed nasal retinal detachment and high reflect dots beneath the retina of the fovea (**f**)

The best-corrected visual acuity (BCVA) was 20/20 in the right eye and 20/200 in the left eye. The intraocular pressure was normal in both eyes. The right eye was essentially normal. Slit lamp biomicroscopy of his left eye showed mixed congestion, dust-like non-granulomatous keratic precipitates (KP), anterior chamber flare (2+) and cells (3+). Iris neovascularization was seen near pupillary margin, and posterior synechias could also be detected (Fig. 1a, b). Fundus examination of the left eye revealed vitreous opacity, retina detached in the nasal side, and several white mass lesions could be faintly seen underneath the retina (Fig. 1c, d).

**Fig. 2:**
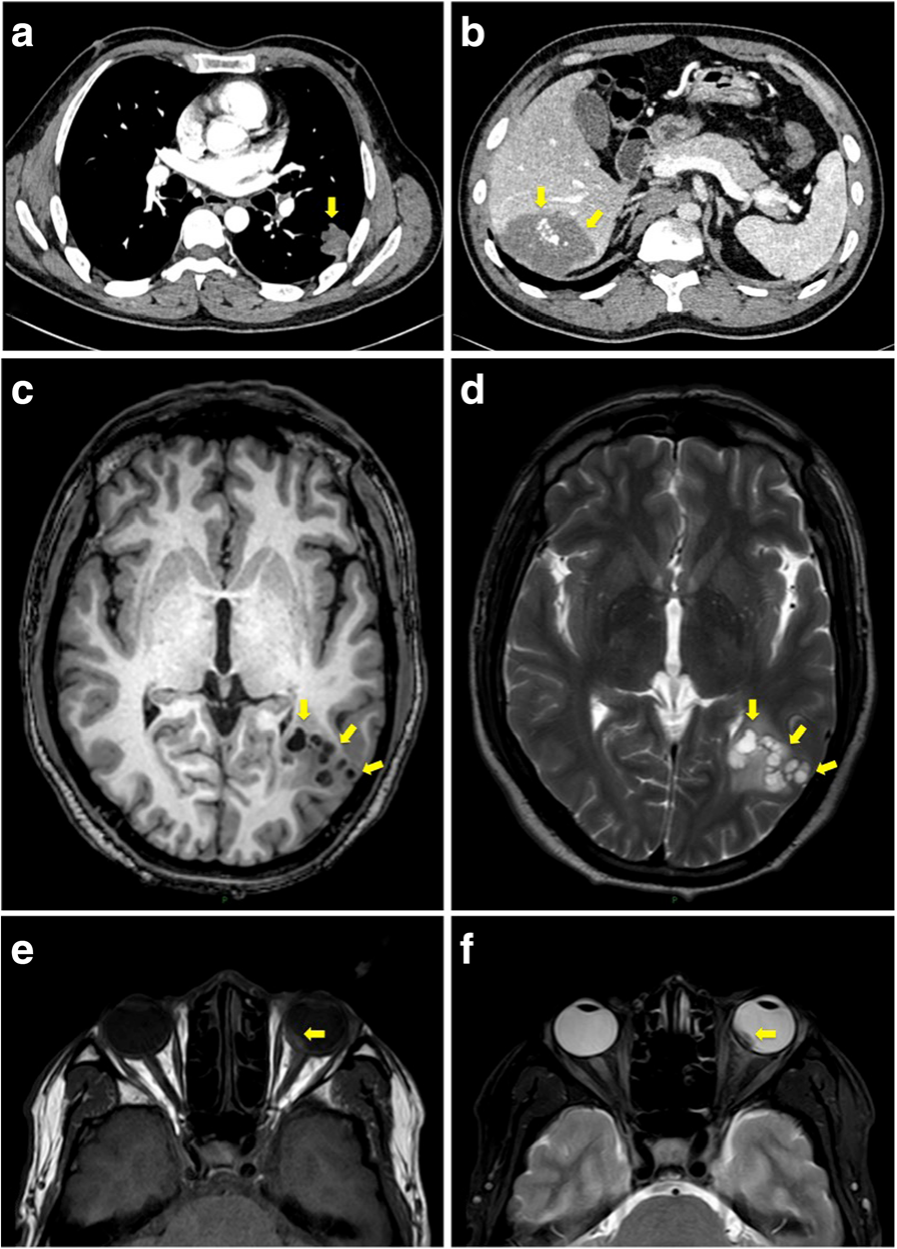
**a** The chest axial enhanced CT image showed an irregular soft tissue density nodule (yellow arrow) in the inferior lobe of the left lung with no obvious enhancement. The size of the lesion was 2.3 cm × 2.5 cm × 2.5 cm (lateral × anteroposterior × superoinferior). **b** Axial abdominal enhanced CT image demonstrated a round hypoattenuating mass in the right lobe of the liver with scattered hyperattenuating foci of calcification (yellow arrows). The mass had no enhancement. Axial unenhanced T1-weighted (**c**) and T2-weighted (**d**) MR images showed multiple cystic lesions (yellow arrows) in the parietal lobe. **e** T1-weighted MR image showed a fusiform isointensity nodule (yellow arrow) in the medial wall of the left eyeball. **f** T2-weighted MR image showed the lesion demonstrates hypointense (yellow arrow)

Ultrasonography revealed retinal detachment and subretinal cystic lesions (Fig. 1e). Spectral domain optical coherence tomography (SD-OCT) scan showed nasal retinal detachment and high reflect dots under macular fovea (Fig. 1f). The chest and abdominal enhanced computed tomography (CT) showed an irregular soft tissue density nodule in the left lung (Fig. 2a, yellow arrow) and a round low-density mass with scattered hyperattenuating foci of calcification in the right lobe of the liver (Fig. 2b, yellow arrows). The brain magnetic resonance imaging (MRI) showed multiple well-defined cystic lesions in the frontal lobe, parietal lobe and occipital lobe (Fig. 2c, d, yellow arrows). Orbital MRI showed a fusiform nodule in the left eyeball (Fig. 2e, f, yellow arrows). Serological test for echinococcosis was weak positive.

**Fig. 3:**
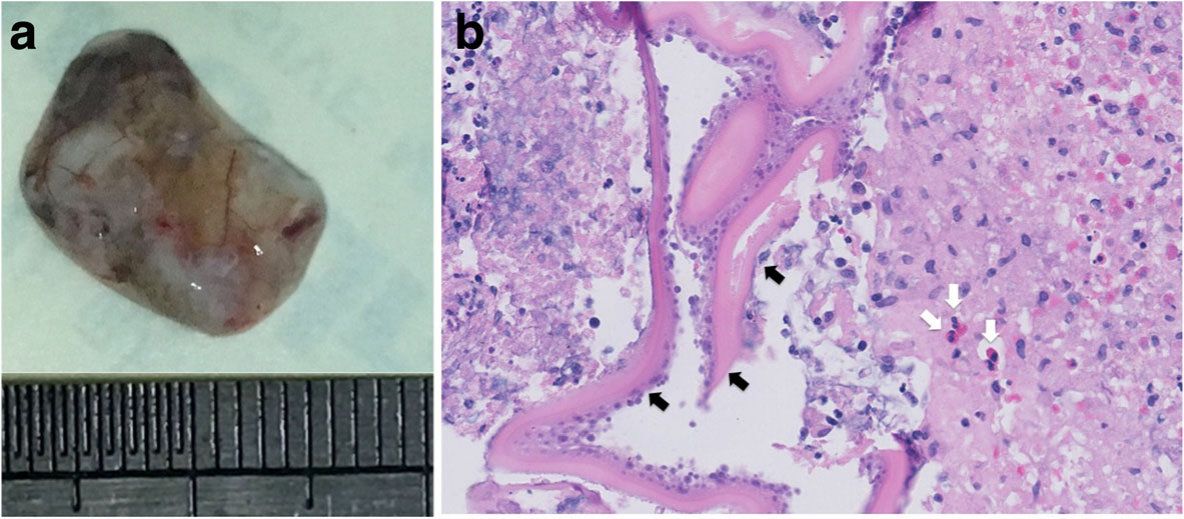
The appearance of the lesion removed in the surgery, part of the retinal attached on the surface (**a**). Hisopathologic slide of the lesion (**b**) showed homogeneous acellular hyaline material (black arrows) and eosinophil granulocytes (white arrows) infiltration in the adjacent necrosis tissue (hematoxylin and eosin stain, original magnification ×400)

According to his agricultural background, cystic lesions in his lung, liver, and brain, and panuveitis in his left eye, mass lesion underneath the retina, the diagnosis of subretinal echinococcosis was made. The patient was treated with 1% prednisolone acetate and pranoprofen eye drops together with oral prednisolone 60 mg per day to control the inflammation. One week later, the inflammation of his left eye turned better. Then, the patient received 0.5 mg ranibizumab and 1 mg triamcinolone acetonide intravitreal injection in his left eye to lessen neovascular and relieve inflammation. Five days after intravitreal injection, the patient received pars plana lensectomy and pars plana vitrectomy. The lesion beneath the retina attached tightly with the retina, so the lesion was removed with the surface retina. It appeared that the lesion was a firm mass tightly adhered together with the posterior sclera and the choroid could not be seen. Laser coagulation was applied at the edge of remained retina, and the vitreous cavity was filled-up by silicon oil. The lesion measured 14 mm × 10 mm (Fig. 3a). Histopathology examination revealed a cyst wall of acellular hyaline material with retinal tissue and eosinophil granulocytes infiltration in the adjacent necrosis tissue (Fig. 3b), confirmed the subretinal echinococcosis diagnosis.

After the operation, the BCVA was 20/200, and the retina was reattached. No complication was observed postoperatively. Further medical treatment with albendazole was suggested, and the patient took the medication in Tibetan local hospitals.

## Discussion

Echinococcosis, also called hydatid disease or hydatidosis, is considered as one of the most dangerous zoonotic parasitic disease worldwide, mainly distributing in Mediterranean regions, Russia, central Asia, China, Australia, South America, and north and east Africa [1, 2]. Echinococcosis occurs as a result of infection by larval stages of taeniid cestodes of the genus *Echinococcus* [3]. Echinococcosis is principally maintained in a dog–sheep–dog cycle. Humans are an accidental intermediate host for this parasite, and normally infected by ingestion of eggs release from dogs or other canids [2]. Echinococcosis is not a common disease in China, but is highly endemic in north-western region and is an important public health problem [2]. Our patient came from rural area of Tibet, where is a high incidence area of cystic echinococcosis. The incidence of human cystic echinococcosis in Tibetan autonomous region was estimated from 1.9 to 155 per 100,000, with a mean incidence of 45.1 per 100,000 [4]. The patient depended on agriculture and animal husbandry for his livelihood, so he had contact with sheep, cattle, and dogs, which made him susceptible to the disease.

The most common affected organs, however, are the liver and the lungs, where 90% of the echinococcal cysts develop [1]. The organism may spread locally and hematogenously to distant sites. CT and MRI are useful methods for human cystic echinococcosis diagnosis. CT and MRI tests demonstrated hydatid cysts in the patient’s liver, lung, and brain. The cysts in these organs usually remain asymptomatic before they grow large enough or rupture to cause secondary infection or allergic reaction [1].

Ocular echinococcosis is exceptionally seldom, and most of the cases involve orbit [5–7]. Orbital involvement takes place in 1%–2% of all hydatid infestation cases [5]. Hydatid cysts in subretinal space are extremely rare [8–10]. Muftuoglu et al. [9] presented a subretinal hydatid cyst case treated with vitreo-retinal surgery. Narang et al. [10] also reported a case of submacular hydatid cyst, pars plana lensectomy and vitrectomy was performed to remove the cyst. In our case, the retina of nasal side was detached and several white mass lesions exist underneath the retina. Ultrasonography confirmed the retinal detachment and subretinal cystic lesions. Pars plana lensectomy and vitrectomy was well planned. The lesion was successfully removed and the retina was reattached after the operation. No complication occurred during and after the surgery and the visual function of our patient was reserved.

Medical treatment with albendazole and mebendazole demonstrated efficacy is useful in the management of patients with hydatid cysts in liver and lungs [1]. Systemic evaluation revealed hepatic, lung and brain involvement in our patient. Since the infestation was asymptomatic, we suggested medical treatment should be applied afterwards.

## Conclusions

This case indicates that subretinal echinococcosis could be a visual-threatening problem in echinococcosis high-risk regions, although the incidence is rare. Vitreo-retinal surgery is an effective method to remove the lesion, and visual function could be retained if the patient is treated properly.

## Abbreviations

BCVA: Best-corrected visual acuity
CT: Computed tomography
KP: Keratic precipitates
MRI: Magnetic resonance imaging
SD-OCT: Spectral domain optical coherence tomography

## References

[1] Mandal S, Mandal MD (2012). Human cystic echinococcosis: epidemiologic, zoonotic, clinical, diagnostic and therapeutic aspects. Asian Pac J Trop Med.

[2] Zhang T, Zhao W, Yang D, Piao D, Huang S, Mi Y, Zhao X, Cao J, Shen Y, Zhang W (2015). Human cystic echinococcosis in Heilongjiang Province, China: a retrospective study. BMC Gastroenterol.

[3] Cadavid Restrepo AM, Yang YR, McManus DP, Gray DJ, Giraudoux P, Barnes TS, Williams GM, Soares Magalhaes RJ, Hamm NA, Clements AC (2016). The landscape epidemiology of echinococcoses. Infect Dis Poverty.

[4] Feng X, Qi X, Yang L, Duan X, Fang B, Gongsang Q, Bartholomot B, Vuitton DA, Wen H, Craig PS (2015). Human cystic and alveolar echinococcosis in the Tibet Autonomous Region (TAR), China. J Helminthol.

[5] Kahveci R, Sanli AM, Gurer B, Sekerci Z (2012). Orbital hydatid cyst. J Neurosurg Pediatr.

[6] Benazzou S, Arkha Y, Derraz S, El Ouahabi A, El Khamlichi A (2010). Orbital hydatid cyst: review of 10 cases. J Craniomaxillofac Surg.

[7] Lentzsch AM, Gobel H, Heindl LM (2016). Primary Orbital Hydatid Cyst. Ophthalmology.

[8] Sen S, Venkatesh P, Chand M (2003). Primary intraocular hydatid cyst with glaucoma. J Pediatr Ophthalmol Strabismus.

[9] Muftuoglu G, Cicik E, Ozdamar A, Yetik H, Ozkan S (2001). Vitreoretinal surgery for a subretinal hydatid cyst. Am J Ophthalmol.

[10] Narang S, Kochhar S, Punia RS, Kumar R, Sambhav K, Sood S (2010). Submacular hydatid cyst: a case report. Retinal Cases Brief Reports.

